# Cells-in-Touch: 3D Printing in Reconstruction and Modelling of Microscopic Biological Geometries for Education and Future Research Applications

**DOI:** 10.3390/bioengineering10060687

**Published:** 2023-06-05

**Authors:** Xavier Fitzpatrick, Alexey Fayzullin, Gonglei Wang, Lindsay Parker, Socrates Dokos, Anna Guller

**Affiliations:** 1 ARC Centre of Excellence for Nanoscale Biophotonics, Sydney, NSW 2052, Australia; 2 The Graduate School of Biomedical Engineering, University of New South Wales, Sydney, NSW 2052, Australia; 3 Institute for Regenerative Medicine, Sechenov First Moscow State Medical University (Sechenov University), 119991 Moscow, Russia; 4 World-Class Research Center “Digital Biodesign and Personalized Healthcare”, Sechenov First Moscow State Medical University (Sechenov University), 119991 Moscow, Russia; 5 School of Natural Sciences, Faculty of Science and Engineering, Macquarie University, Sydney, NSW 2109, Australia; 6 Macquarie Medical School, Faculty of Medicine, Health and Human Sciences, Macquarie University, Sydney, NSW 2109, Australia

**Keywords:** 3D printing, microscopy, CAD, FDM, cell shape, cytoskeleton, tactile education, data visualization, modeling, Materialise Mimics, Cito-3DP

## Abstract

Additive manufacturing (3D printing) and computer-aided design (CAD) still have limited uptake in biomedical and bioengineering research and education, despite the significant potential of these technologies. The utility of organ-scale 3D-printed models of living structures is widely appreciated, while the workflows for microscopy data translation into tactile accessible replicas are not well developed yet. Here, we demonstrate an accessible and reproducible CAD-based methodology for generating 3D-printed scalable models of human cells cultured in vitro and imaged using conventional scanning confocal microscopy with fused deposition modeling (FDM) 3D printing. We termed this technology CiTo-3DP (Cells-in-Touch for 3D Printing). As a proof-of-concept, we created dismountable CiTo-3DP models of human epithelial, mesenchymal, and neural cells by using selectively stained nuclei and cytoskeletal components. We also provide educational and research context for the presented cellular models. In the future, the CiTo-3DP approach can be adapted to different imaging and 3D printing modalities and comprehensively present various cell types, subcellular structures, and extracellular matrices. The resulting CAD and 3D printed models could be used for a broad spectrum of education and research applications.

## 1. Introduction

Additive manufacturing (AM), commonly termed 3D printing [1], is a methodology of physical reconstruction of three-dimensional structures and complex geometries from digital models of these objects formed (in a core concept, and in contrast to the traditional subtractive or formative manufacturing approaches) by layered deposition of the material [2]. The success of AM may be attributed to its affordability, flexibility, safety, and efficiency compared to more traditional manufacturing processes [3]. The most common modalities of 3D printing, in order of increasing spatial resolution capacity, include powder bed fusion (e.g., selective laser sintering), inkjet printing, stereolithography, and fused deposition modeling (FDM) [1]. The availability of affordable FDM desktop 3D printers and open-access processing software has made this technology truly global and friendly for entry-level consumers [4].

The workflow for most AM technologies includes (1) computer-aided design (CAD) as a process of transforming the imaging data into a digital model representation of a 3D object, (2) model post-processing, or “slicing”, and (3) printing [5]. There are several CAD options for reconstructing images into digital 3D models in a stereolithographic format (STL), both commercial and open-access [6,7], as well as many commercially available 3D printers that use STL format files for FDM. Further to this, various materials can be used for 3D printing, offering a range of textures, strengths, and extrusion properties. The most common FDM printing materials are acrylonitrile butadiene styrene (ABS) and polylactic acid (PLA) filaments. These plastics are compatible with various FDM 3D printers and are similar in their performance and affordable cost [8], while PLA has a much better safety profile than ABS [9].

Medicine and bioengineering are promising areas of AM applications. For example, images obtained by computer tomography (CT) and magnetic resonance imaging (MRI) have been successfully utilized to 3D print individualized prosthetics [10,11], implants, and pro-regenerative scaffolds [2,12]. It is important to note that modern 3D printers have a higher isometric resolution (~100 µm) than CT or MRI scans (i.e., the CT pixel size is 0.15–0.5 mm and interslice distance is 0.4–1.0 mm), allowing a very detailed reproduction of various macroscopic (cm scale) body structures [13]. Three-dimensionally printed patient-specific anatomical models provide a particularly excellent opportunity for pre-interventional tactile and visual appreciation for better surgical planning, including choosing the appropriate implantable devices, and improvement of treatment outcomes [2,14,15,16,17,18]. Three-dimensionally printed tissue models help link the application of AM to tactile visualization of living structures in the human body [19].

Recently, AM has expanded into the realm of microscopy, both from the instruments’ building [6] and the biological objects’ physical reconstruction aspects [4,5,20,21,22,23,24]. In general, 3D printing of the microscopy-imaged structures follows the standard CAD-to-AM workflow. However, in contrast to the reconstruction of the objects visualized by CT and MRI, microscopy-to-3D printing relies on the source images with spatial resolution, which is higher than those achievable [13] by modern 3D printers. The spatial resolution for light diffraction-limited imaging modalities is approximately 1 µm, while electron microscopy and super-resolution techniques allow the depiction of nanoscale features. Therefore, to achieve biologically accurate modeling, microscopy-to-3D printing workflow requires an extra step of rational rescaling the CAD model into a tactile-accessible size.

To date, the models of four classes of microscopic biological objects, such as (i) whole cells of mammalian and invertebrate origin, (ii) pollen, (iii) parts or clusters of plant cells, and (iv) whole embryos of small laboratory animals have been 3D printed (Table A1 in Appendix A). Five imaging modalities were employed for the generation of the source images, including stimulated emission depletion microscopy (STED) and serial electron tomography based on transmission electron microscopy (TEM), multiphoton microscopy, lightsheet microscopy, and scanning confocal microscopy (SCM). Expectably, SCM is emerging as the most accessible imaging method that allows 3D virtual reconstructions, while FDM is becoming the most used printing modality in microscopy-to-3D printing modeling of biological objects.

Despite the fast development of this approach, it still has some substantial knowledge and methodological gaps. (1) Firstly, no microscopy-to-3D printing models of mammalian cells that form solid tissues have been demonstrated yet. Additionally, there is still no comparative presentation of different types of cells/tissues of origin, health and disease states, embryonic origins, and functional polarization in the 3D printed form. (2) Next, despite using different contrasting methods, no multiplexing of contrast agents has been employed in the published examples of 3D printed models of biological microstructures. (3) Finally, none of the published protocols allowed interactive dismountability. In simple words, this option allows “assembling” or “disassembling” the “cell” which could provide an engaging interactive stimulus for better integrative 3D printed models in educational and research contexts.

Here, we address the indicated challenges and present our proof-of-concept study together with the practical protocols for the methodology which we termed CiTo-3DP (**C**ells-**i**n-**To**uch for **3D P**rinting) for producing 3D PLA prints from SCM serial images (z-stacks) of micrometer scale biological objects. Using this approach, we created 3D-printed models of epithelial, mesenchymal, and neural human cells. These cell types are representative of solid tissues from different embryonic origins and have fundamental morphological differences that define the respective phenotypes. Using fluorescent contrasting agents, we visualized and printed subcellular structures. These structures include the nuclei and two types of cytoskeletal elements (f-actin stress fibers and contractile α-smooth muscle actin (α-SMA)) with a digital reconstruction of the cell surface shape. To enhance interactivity, we made our models dismountable. In addition, our work provides rich research and educational context of the presented workflow (Appendix A.1). The diversity of future applications for the CiTo-3DP approach is also discussed.

## 2. Materials and Methods

### 2.1. Study Design

**Figure 1:**
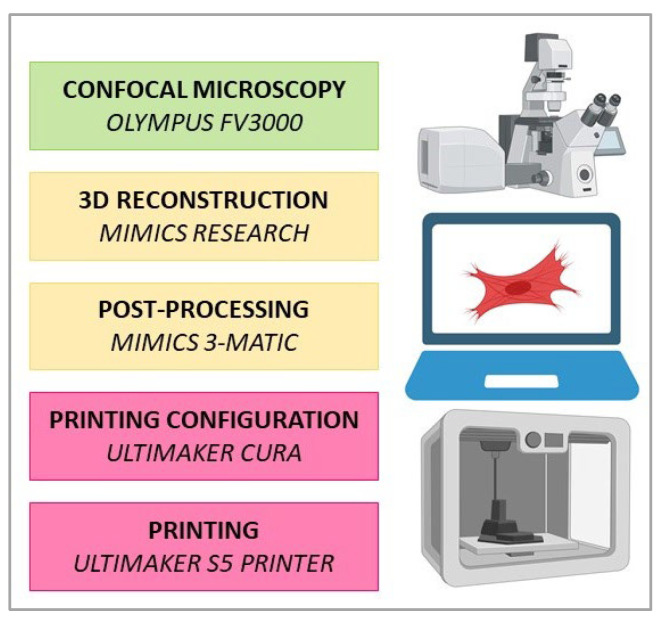
The workflow applied in the current study. Z-stacks of 2D cell images were acquired with an Olympus FV3000 confocal laser scanning microscope. Subsequently, CAD models of the cells were generated using Mimics Research software v21.0. Post-processing was performed using Mimics 3-matic. Finalization of the model for printing was conducted using Ultimaker CURA. PLA models were printed with an Ultimaker S5 printer.

The following method was designed to generate 3D cell reconstructions from immunofluorescent confocal z-stack images of adherent in vitro cultured cells and optimized for 3D printing on commercially available FDM printers. The workflow applied in the current study is schematically shown in Figure 1. Three cell types were used for the proof-of-concept experimentation of the CiTo-3DP methodology, including human epithelial, mesenchymal, and neuronal cells. The model of an epithelial tissue cell was based on the images of the linear cells PANC-1, which are representative of the parenchyma of the pancreas in the state of malignancy (pancreatic adenocarcinoma). The mesenchymal phenotype was shown using primary healthy fibroblasts of skin derma (human dermal fibroblasts, HDFs). A model of a neuron was created based on the images of cell line SH-SY5Y, which is representative of neuroblastoma. The detailed technical notes for the workflow presented here are provided in Appendix B.1.

### 2.2. Cell Culture and Staining Procedures

The HDFs (#106-05A, Sigma-Aldrich, Sydney, Australia) were cultured in fibroblast growth medium (#116-500, Sigma-Aldrich) according to the recommended protocol [25]. PANC-1 cells (CRL-1469™, ATTC) were cultured in Dulbecco’s modified eagle’s medium (#11995, DMEM, high glucose, pyruvate, Thermo Fisher, North Ryde, Australia) with 10% fetal bovine serum (FBS) (#16000044, Thermo Fisher), 1% antibiotic-antimycotic (#A5955, Sigma-Aldrich, Australia) and 1% L-glutamine. The neuron-like cells SH-SY5Y were cultured in DMEM supplemented with 10% FBS, 2 mM L-glutamine, 100 IU/mL penicillin, and 100 mg/mL streptomycin. All cell types were cultured in Corning T-75 cell culture flasks (#CLS430641, Sigma-Aldrich) at 37 °C with a 5% CO_2_. Cells were routinely passaged twice per week (after reaching 70–80% confluency) via detachment with Trypsin/EDTA solution (#T3924, Sigma-Aldrich).

For the imaging of the subcellular structures, cells were further cultured in 35 mm coverslip-bottomed culture dishes (#81156, Ibidi, Gräfelfing, Germany) overnight, and then washed with phosphate-buffered saline (PBS; #D8537, Sigma-Aldrich) and fixed with 10% neutral buffered formalin (#HT501128, Sigma-Aldrich) for 20 min at room temperature. Following two washes with PBS, cellular nuclei were stained with NucBlue™ Fixed Cell ReadyProbes™ Reagent (DAPI) (#R37606, Thermo Fisher), and the f-actin filaments of the cytoskeleton were stained with Phalloidin-TRITC (#P1951, Sigma-Aldrich). The HDF samples were also stained with the mouse anti-human α-SMA monoclonal antibody (#A2547, Sigma-Aldrich) and the donkey anti-mouse secondary antibody Alexa Fluor Plus 647 (#R322787, Invitrogen). Stained cells were washed and stored in PBS at 4 °C with protection from visible light until imaging (24–48 h).

### 2.3. Image Acquisition and 3D Reconstruction

Cells were imaged using an Olympus FV3000 confocal laser scanning microscopy system (Olympus, Tokyo, Japan). The confocal microscopy settings and image parameters used in this study are shown in Table A2 and Table A3 in Appendix A.

**Figure A1:**
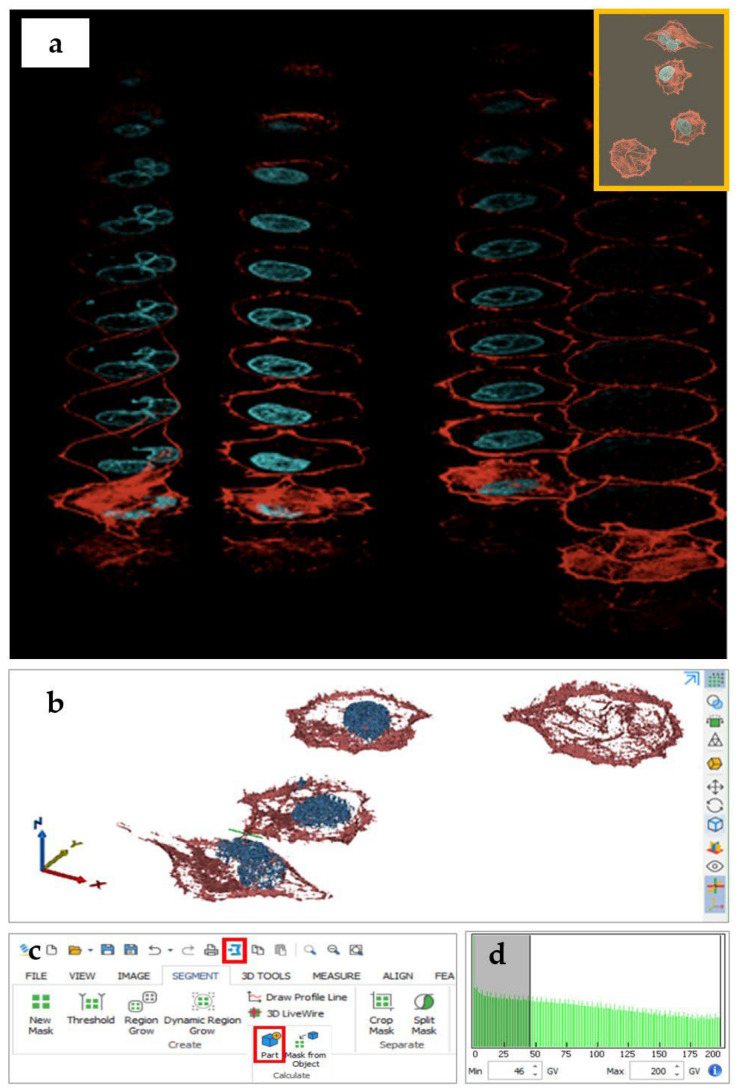
The 3D reconstruction workflow. (**a**) Individual “slices” of the confocal microscope image of PANC-1 cells with the nuclei stained by DAPI (blue) and cytoskeletal f-actin filaments stained with phalloidin-TRITC (red); insert highlighted in yellow shows a maximum intensity projection image of the “sliced” four PANC-1 cells; (**b**) segmented PANC-1 cell reconstructions generated from z-stack images (blue—nucleus, brown—f-actin cytoskeleton); (**c**) a screenshot of Materialise Mimics Research software toolbar with the “Create” part and part of “Export” tools highlighted; (**d**) thresholding grey value (GV) histogram.

The TIFF z-stack images were imported into a biomedical image segmentation software, Mimics Research 21.0 (Materialise, Leuven, Belgium), which is commonly used in 3D macro-anatomical analysis of DICOM images (Figure A1 in Appendix B). It should be noted that image quality is the most important contributor to reconstruction accuracy. On import, image aspect and scale, in nm or µm, were validated against the coronal, axial, and sagittal coordinate axes.

The main enabling tool used for 3D reconstruction of images was thresholding. The following workflow was applied: thresholding (tool: segment > threshold; or tool: segment > dynamic region grow) inputs grey-scale, or “Grey Value” (GV), pixel-intensity maxima and minima, allowing for 3D image segmentation into new masks appearing in the software’s project management and 3D previewer windows. New masks are comprised of tessellated mesh surfaces, wrapped around individual or adjacent image pixels. In this way, imported image stacks were organized into 3D reconstructions of isolated cellular components. Alternatively, cellular components were separated by splitting the mask (tool: split mask). Following this, masks were cropped (tool: segment > crop mask; or tool: segment > region grow) to include only the information required. In cell biology, single cells or smaller cellular clusters may be segmented in this way.

Due to the nature of fluorescent staining and confocal microscopy imaging, as well as the nature of the imaged subcellular structures, where the cytoskeleton plays the role of the tension-bearing element for the outer cell membrane, there could be several holes in the reconstructed cell membrane surface. Therefore, the surfaces of the segmented masks were expanded by filling (tool: segment > smart fill) and brushing in the individual 2D images (tool: segment > smart fill > local fill). Any reconstruction errors that are inconsistent with the imaged biology, which may arise due to image resolution and thresholding, were edited by highlighting the respective region (tool: segment > edit masks). The next step was optimization of the obtained 3D reconstruction for printing (post-processing).

### 2.4. Post-Processing

**Figure 2:**
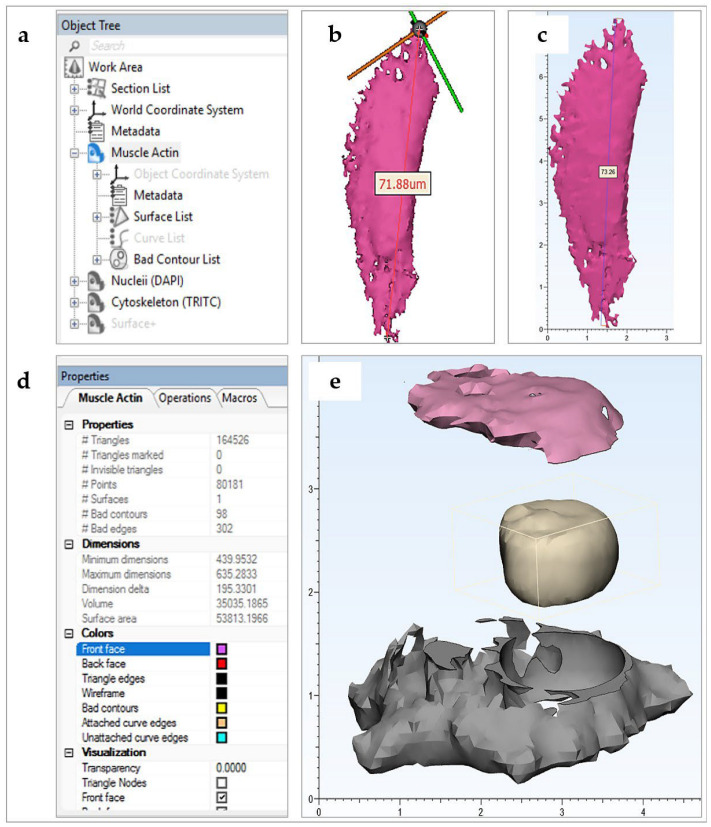
Elements of the post-processing workflow. (**a**–**d**) Post-processing illustration applied for creation of the HDF model; (**a**) 3-Matic Object Tree displaying imported HDF geometries (α-SMA (Alexa Fluor 647), nuclei (DAPI), cytoskeleton (TRITC)) and software metadata; (**b**) length and scale verification of HDF nuclei (DAPI) in Mimics Research (L; um) and (**c**) in Mimics 3-Matic Research (R; cm); (**d**) a screenshot of the selected object properties tab for an HDF a-SMA muscle actin (Alexa Fluor 647) part in the Materialise 3-Matic user interface; (**e**) Post-processed PANC-1 cell model (cm) designed as a dismountable set for greater interactivity. Note the nucleus geometry was Boolean subtracted from the cytoskeleton with positive clearance factor for post-printing compatibility. The cytoskeleton geometry was also split into upper (shown in pink) and lower (shown in grey) parts.

With the surfaces segmented and ready for post-processing, the relevant masks were converted into meshed geometries or parts. The parts (tool: segment > part) were exported into *Mimics 3-matic* software v13.0 (Figure 2a).

*Mimics Research* works in the validated image scale, but *Mimics 3-matic* is constrained to operate in mm, with actual scale stored in memory. Although this scale transformation is automatic within the Materialise (Leuven, Belgium) software package, it was validated by measurement of key lengths in both software packages (tool: measure > distance) (Figure 2b,c).

Imported parts, displayed in the software Object Tree, were color coordinated (tool: object tree > object properties > colors) and aligned (tool: align) for improved workflow. To view object interiors, a viewing plane was defined and translated through the object (tool: object tree > section list > standard section > position step size). This proved useful in examining the compliance of meshed objects to the imaged biology.

At this stage, meshes were representative of surfaces only, making them impossible to print with extruded filament of a non-negligible thickness. In practice, printing surfaces with thicknesses greater than or equal to 1 mm generate stable models, although this may vary with printing material and printer used. In *Mimics 3-Matic*, meshes were uniformly offset (tool: design > uniform offset > solid) by a minimum distance of 1 mm, with the solid fill option checked. Next, the models were smoothed (tool: fix > smooth; or fix > reduce; or fix > wrap; or finish > local smoothing; or remesh) to simplify tessellation and hence reduce printing time and cost (Figure 2d). This action is also known to improve the likelihood of printing success without sacrificing significant resolution.

To improve the educational interactivity of the models, a range of editing tools are available in the software. In the presented CiTo-3DP methodology, PANC-1, and neuronal SH-SY5Y cell models were trimmed (tool: finish > trim > preserve inner and outer) to split the cytoskeleton component in two. Further to this, the nuclear component was removed, with a positive clearance factor in mm, from the cytoskeleton, allowing it to fit neatly inside the split parts (tool: design > local Boolean > subtraction) (Figure 2e). The same approach was utilized to separate two cytoskeleton components (f-actin and α-SMA) and nuclei in the fibroblast models. If components are to be joined together by design slots or joints, a datum plane must be defined (tool: design > create analytical primitive > create datum plane), such that the relevant geometry may be cut (tool: design > cut) about the plane and designed for fitting (tool: design > create primitive; design > Boolean union). Note that compliance and compatibility must be carefully considered for part-fitting.

To finalize meshed geometries, the software automatic mesh corrector algorithm (tool: fix > fix wizard > follow advice) was used. After this, the respective objects, now optimized for 3D printing, were exported as STL files into relevant pre-printing software. In our methodology, these STL files were opened in *Ultimaker’s* pre-print software *CURA* v4.7.0.

### 2.5. Printing

**Figure A2:**
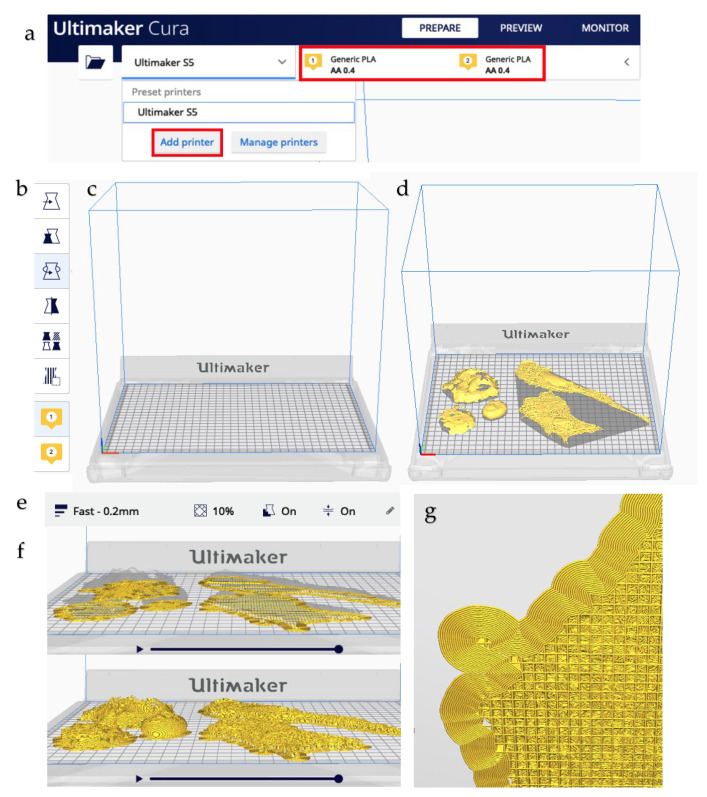
The 3D printing procedure. (**a**) Configuring the Ultimaker CURA v4.7.0. software. Printer and material configuration tools are highlighted. (**b**) Pre-print editing toolbox. (**c**) Virtual Ultimaker S5 printer. (**d**) PANC-1 and HDF cell .stl files imported into software. (**e**) Print settings. (**f**) Post-slicing virtual printing check. (**g**) Base plate support structures. Note that these are removed after printing.

*CURA* is an open-access software, allowing users to import STL files into a virtual 3D workplace of the specific printer chosen for printing (Figure A2 in Appendix B).

Prior to opening the relevant STL files, *CURA* was configured to the printer used (tool: add printer). A wide range of pre-set printer configurations from *Ultimaker* (Geldermalsen, Netherlands) and other 3D printing companies is included in the software. The size of the printing bed, the type and number of extruders and the material used for extrusion were all defined, as were the slice orientations, layer thickness, infill, and settings for the printing of supports. The software also provided a 3D virtual preview of the print process to visualize the model as it would be printed, allowing further edits and refinements to the print strategy prior to actual printing. The relevant STL models, which use the 3-matic mm scale, were imported and adjusted to best fit on the printing bed. Any changes to scale were noted.

As the presented workflow was used as a proof-of-concept, the printing configurations were selected for fast PLA printing, which correlates to a printed layer height of 0.2 mm. Notably, printing speed is directly related to layer height in millimeters and hence determines the quality or resolution of the final print. Shell thickness, or the number of horizontal layers in each shell, affects the final stability of the print, as does infill. A triangular infill of 10% was used for fast printing. Supports were generated, followed by object slicing, which determined the exact printing path the extruder would follow.

The printing configuration and path followed determine speed of the print and the amount of material used. The sliced objects were exported directly into the printing hardware. Printing was initially observed to check for common 3D printing errors such as extruder clogging or poor build plate adhesion. Once printing was finished, models were allowed to cool and then removed from the build plate. Printing supports were removed manually. As such, a 3D reconstruction of complex cell geometry, imaged using confocal microscopy, was printed.

## 3. Results

The cell types selected for modeling differed significantly in their morphometric characteristics (Table 1). Further 3D modeling using CiTo-3DP methodology allowed reliable reproduction of the key features of the studied cells. The resolution of each print was calculated using the printing scale and layer height (Table A4 in Appendix B).

**Figure 3:**
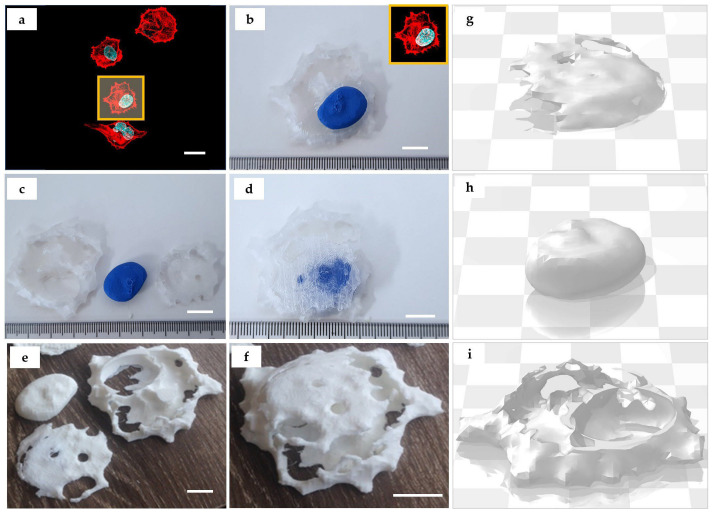
Three-dimensionally printed model of an epithelial cell (PANC-1). (**a**) Maximum intensity projection of z-stack of 16 fluorescence confocal images obtained across the section thickness of 15 μm. The area highlighted in yellow indicates the individual cell selected for the 3D printing. Staining: cell nuclei—turquoise (DAPI), and f-actin cytoskeleton—red (phalloidin-TRITC). Scale bar: 25 µm; (**b**–**f**) details of the 3D printed model of a PANC-1 cell: (**b**) correspondence between the original image (insert highlighted by yellow frame) and the printed model. The image depicts a reconstructed lower surface of cell which is printed using translucent filament, and the nucleus which is printed using blue filament; (**c**) the model allows for disassembly in 3 parts (translucent lower and upper parts of the reconstructed cytoskeleton and cell surface, and blue nucleus); (**d**) re-assembled 3D printed model of PANC-1 cell: note the enclosure of the blue nucleus in the translucent parts of the reconstructed cell surface. (**e**–**f**) Three-dimensional printing of the PANC-1 cell model using a single-color PLA filament (white). (**e**) Dismountable PANC-1 cell model in the “open” state—with nucleus removed from the two parts representing the surrounding f-actin cytoskeleton and the stretched cell membrane. The “niche” formed by the cytoskeleton f-actin filaments that surround the nucleus in the cell is clearly visible in the top right fragment. Scale bars (**b**–**f**): 1 cm (printed model): 10 µm (original); (**g**–**i**) the STL 3D models of the upper (**g**) and (**i**) lower parts of the cytoskeleton and reconstructed cell surface, and the nucleus (**h**) used for printing of the models shown in (**b**–**f**).

As follows from Table 1, the epithelial cell representative for the pancreatic adenocarcinoma (PANC-1) showed a compact phenotype, compared to fibroblasts. PANC-1 cells featured a round cell shape without long protrusions, higher density of f-actin at the outer cell borders (cortical localization), and centrally or slightly eccentrically located roundish nuclei (Figure 3a). Note that the shape of the nucleus was irregular, in contrast to the common perception. The pancreatic cell model was 3D printed in colored and single-color (white) versions and made dismountable (Figure 3b–f). In this model, the post-processing operations allowed the reconstruction of the internal space and the outer cell shape based on the configuration of f-actin cytoskeleton filaments. Interestingly, the PANC-1 cell model revealed the existence of the specific “niche” formed by f-actin filaments around the nucleus, which was not appreciable in confocal microscopy images, while it became clearly visible during virtual 3D conversion of the confocal z-stacks into STL files (Figure 3g–i).

**Figure 4:**
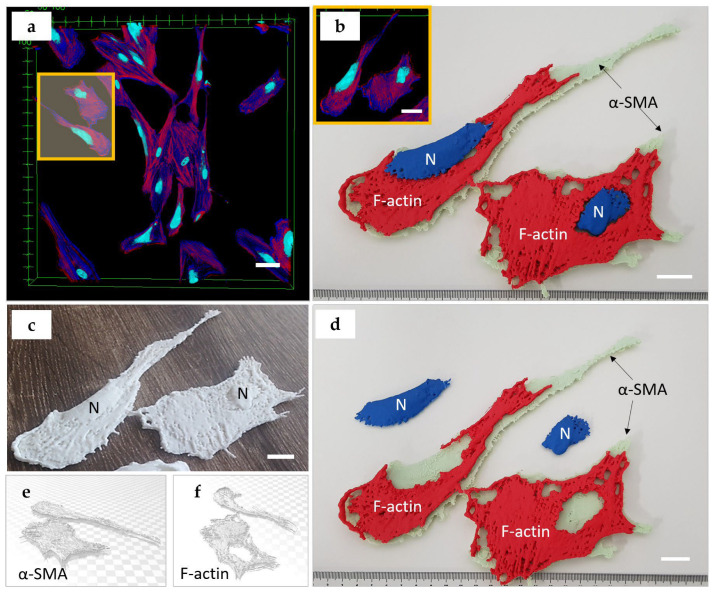
A 3D printed model of mesenchymal cells (HDFs). (**a**) Maximum intensity projection of z-stack of 33 fluorescence confocal images obtained across a section thickness of 16 μm. The area highlighted in yellow indicates two cells selected for the 3D printing. Staining: cell nuclei—turquoise (DAPI), α-SMA actin fibers that surround the nuclei (blue, secondary antibody conjugated with AlexaFluor 647), and the f-actin stress fibers located at the cortical surfaces of the cells (red, phalloidin-TRITC). Scale bar: 100 µm; (**b**–**d**) 3D printed model of HDF cells: (**b**) shows correspondence between the original image (insert highlighted by yellow frame) and the printed models. The reconstructed f-actin cytoskeleton is printed using red filament, α-SMA actin fibers are printed using mint-colored filament, and the nucleus is printed using blue filament. The insert shows the cells selected for printing from the original image; (**c**) the single color (white) non-dismountable version of the 3D printed model of HDFs; (**d**) the color model allows for disassembly in 3 parts (2 types of cytoskeleton fibers, and the nucleus); scale bars (**b**–**d**): 2 cm (printed model): 100 µm (original); (**e**,**f**) STL 3D models of α-SMA (**e**,**f**) f-actin components of the cytoskeleton.

A mesenchymal cell phenotype was presented by primary fibroblasts derived from human skin derma. These cells showed typical spindle-like and relatively flattened cell bodies, with centrally located nuclei of various shapes. Notably, the HDFs cultured on stiff plastic surfaces also possessed α-SMA cytoskeletal filaments, which are a specific marker of differentiation into a contractile fibroblast phenotype (myofibroblasts), known to be responsible for fibrotic (scarring) processes (Figure 4a). In the 3D printed model, we reconstructed two HDFs that were contacting each other in cell culture. We produced a multicolored dismountable model, which included two parts of cytoskeleton (red PLA filament was used for modeling of f-actin, and the mint-colored filament was applied for α-SMA) and the nuclei were printed in blue color (Figure 4b,d). Interestingly, the nuclei of HDFs had a complex, slightly flattened shape, with delicately branched edges. The cellular f-actin filaments also surrounded the nuclei as was observed in the epithelial cell model. In contrast, the α-SMA fibers did not exhibit spatial coordination with the nuclei. Next, we demonstrated solid 3D-printed models of the same fibroblasts in a white color (Figure 4c) to emphasize the integration of the nuclei and two types of actin in the cytoskeleton. In Figure 4e,f, the 3D STL models for f-actin and α-SMA are shown.

**Figure 5:**
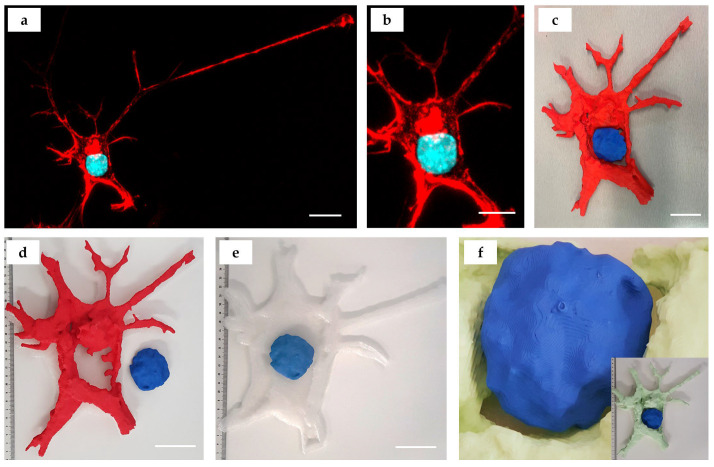
A 3D printed model of a neuronal-like cell (SH-SY5Y). (**a**) Maximum intensity projection of z-stack of 36 fluorescence confocal images obtained across the section thickness of 10 μm. Staining: cell nuclei—turquoise (DAPI), and f-actin cytoskeleton—red (phalloidin-TRITC). Scale bar: 10 µm; (**b**) segmented fragment of the neuron image (z-stack shown in (**a**)) used for modeling and printing, scale bar: 5 µm; (**c**) 3D printed model of an SH-SY5Y cell depicted in (**a**,**b**); the f-actin cytoskeleton is printed using red filament; the nucleus is printed with blue filament; (**d**) disassembly of the neuron model is in 2 parts (red f-actin cytoskeleton and blue nucleus); (**e**) reconstruction of the lower cell surface (3D printed using translucent filament) with a superimposed blue nucleus model; (**f**) enlarged view of the surface of the cell nucleus model located within the mint-colored f-actin “niche”. Insert shows 3D printed model of the same neuron as in (**a**–**e**) that was printed in mint (f-actin) and blue (nucleus) colors. Scale bars in (**c**–**e**): 5 cm (printed model): 5 µm (original).

Neuronal-like differentiated SH-SY5Y cells were approximately three times smaller than the epithelial cells (PANC-1). They featured polygonal cell body shapes containing round-shaped nuclei with multiple axonal or dendritic protrusions (Figure 5a). For 3D printing, we segmented a central part of a single neuronal-like cell (Figure 5b). The staining pattern (red color for f-actin cytoskeleton and turquoise/blue for nuclei was reproduced in the 3D printed model that allowed for dismountability (Figure 5c,d). We applied a post-processing protocol to reconstruct the lower cell surface based on the f-actin fibers cytoskeleton configuration (Figure 5e). We also demonstrated a 3D printed model with an alternative color scheme (with mint-colored f-actin and blue nucleus) and revealed the complexity of the cellular nucleus surface shape (Figure 5f).

## 4. Discussion

The microscopy-to-3D printing concept allows upscaling the unseen world of microscopy into perceptible matter. This provides researchers and educators with a tool to present their discoveries and teaching content at a more comprehensible scale, making it easier to communicate complex biomolecular subjects.

In the current study, FDM printing technology was utilized to produce CAD-generated 3D reconstructions of confocal microscopy whole-cell imaging data. We utilized FDM 3D printing in our CiTo-3DP methodology due to its ease of use, speed, considerable commercial availability, and affordable operation. The FDM technology has reasonable resolution capabilities, with most commercial products able to print to actual resolutions, or extrusion layer heights, of down to 100 µm [13]. FDM 3D printing devices are also capable of printing other materials with varying physical attributes, such as flexibility, strength and transparency, and colors, although safety and printer compatibility need to be considered. We used not only white PLA material but also demonstrated that the multicolored and transparent materials can be adapted to our proposed CiTo-3DP protocol in a way similar to published prototypes [13,20,26]. Additional finishing of the models for perception enhancement can be performed, for example, by coating them with silicone rubber as shown elsewhere [8].

In our CiTo-3DP workflow, an Ultimaker 3D printer was chosen for model production. The advantage of using Ultimaker hardware is its ease of use, commercial availability, affordability, and compatibility with pre-print software CURA v4.7.0. The latter included a virtual 3D visualization of the print process itself, allowing prior adjustment of various print settings such as print slice orientation, infills, and printing of supports. For more complex geometries, however, greater control and editing of the printing path could be an advantage in specific cases, although CURA has the advantage of being readily available. In saying that, due to the competitive market, we regarded other 3D printing hardware and software as comparable and easily interchangeable with the presented workflow.

The current study represents a “proof-of-concept” technical note limited to the translation from the confocal images of the cells to the tactile models using FDM as the most accessible and affordable AM method. At the same time, more complex AM technologies such as SLS and two-photon 3D printing are indeed becoming more readily available. These advanced approaches offer greater precision, potentially making them better suited to the field of microscopy, where model upscaling, image resolution, and printing accuracy are vitally important. However, currently, they still appear less accessible and more expensive to entry-level users when compared with FDM 3D printing, and hence will likely experience less uptake into new industries. We envisage that in the future, the proposed CiTo-3DP methodology can be easily expanded and customized to merge with not only various additional staining methods (e.g., immunocytochemistry, organelle trackers), and high-resolution microscopy modalities, such as electron microscopy or super-resolution microscopy, but also to the light-curing 3D printing workflows (e.g., 2-photon nanoparticles-aided polymerization [27]). This will allow the rapid creation of cellular models and subcellular structures of very high resolution and structural fidelity that potentially may be used in further bioengineering applications (e.g., preparation of tissue engineering scaffolds).

The future of image processing and AM utilization in cell biology and related disciplines is promising. Various steps have been taken toward integrating image-based model simulations into common practice. Togni et al. [28] showed the efficacy of using finite-element method (FEM) multi-physics modeling software in undergraduate biology education, whilst Tang et al. [29] compared the biomechanical heterogeneity of living cells as measured by atomic force microscopy and finite-element simulation. Notably, both used generic computer-defined geometries. To implement this into a 3D printing workflow, further steps would be required to better define the objects. Inspecting surface mesh quality, generating a volume mesh, and validating against the imaged biology, would be required as a minimum to ensure accurate modeling. A range of finite element method and computational fluid dynamics (CFD) software, such as ANSYS, COMSOL Multiphysics, or even Materialise, are available, providing the file types and sizes that are transferable between software.

Another promising technology entering this field is virtual reality. Virtual reality visualizations require similar image-processing analysis and hence provide equivalent educational benefits to students, all whilst negating the need and hence the cost of 3D printing. This too, has seen limited uptake in cell biology education. In the study by Cali et al. [30], virtual reality was used to visualize and aid quantitative analysis of reconstructed glial and neuronal cells.

FDM 3D printed cellular and subcellular models have the potential to be used both as a visual aid, as described, and as a quantitative tool. This is of particular interest in the fields of bioengineering, computational biology, cellular and tissue morphometrics, and developmental biology. Analysis of morphogenetic behavior of living tissues has to date proven instrumental in biology-related fields [31], and 3D image reconstruction and FDM-printing pose as additional analytical tools. In the presented methodology, the clear differences between the PANC-1 (epithelial), HDF (mesenchymal), and SH-SY5Y (neuronal) phenotypes were revealed using 3D printed models and were shown across several cellular structures. Image processing and reconstruction of 3D geometries make basic morphometric measurements, such as cellular diameter, shape, height, and surface area easier to acquire. Additionally, the segmentation of various cellular structures allows intra-cellular comparisons to be made. FDM-printing models with the same material would also provide data on cellular volumetrics. That is, the amount of material required to print cellular structures of different cell types could be used as a comparative measurement. To improve image quality, finer voxel dimensions are recommended. Clearly, the presented workflow could be utilized for quantitative morphometrics with minimal adjustments.

Finally, introducing 3D models for the presentation of experimental results in biological systems is a part of the trend to put discoveries in more translatable models. This is especially instrumental for research conducted on cellular and tissue levels since both cell microscopy and pathology lose the volumetric perspective. We hope that additive technology models can contribute to a better understanding of the spatial profile of tissues, accelerating research in matrix biology and mechanotransduction.

The development of this approach has the potential to further revolutionize science education, by providing a strong nexus between laboratory skills, computational analysis, and communication of results [13]. This method has already been utilized in advancing the analysis of biomolecular data sets in the teaching of complex chemical molecular structures [26]. It is reasonable to suggest that AM technology applied for the reconstruction of micron-scale biological objects can contribute to knowledge generation advancement in life and material sciences, engineering, and medicine.

Notably, 3D printing also offers an innovative and feasible way of introducing tactility into the educational curriculum, resulting in greatly improved learning outcomes by 3D printed models as tactile data visualizations [32]. For example, detailed 3D-printed anatomical models of prosected organs allow the replacement of several expensive and labor-intensive processes used in medical education [13]. In fact, 3D-printed replicas provide a physical interface through which users can directly interact with the source data and obtain difficult scientific and engineering concepts in a more accessible way. Such an approach reduces the cognitive load and improves knowledge translation. Three-dimensionally printed models also can enhance learning experiences for visually impaired and disabled students and for students with special needs [32].

Three-dimensional printing does not come without limitations. Firstly, and most importantly, model quality is intrinsically dependent on microscopy image quality. That is, the mode of image acquisition has a direct impact on the final quality of results. Available computational power should also be considered regarding any increase in imaging resolution. Furthermore, using our proposed CiTo-3DP methodology, it is difficult to visualize smaller cellular structures, such as ribosomes, even after upscaling. Beyond the image quality and microscope resolution, it is also constrained by the resolution capacity of FDM printing and the spatial limitations of commercial printers. That is, increasing scaling factors to visualize more detailed biological structures would hinder project time, cost, and model ease-of-interactivity.

Nevertheless, the CiTo-3DP methodology we have outlined here is highly transferrable and flexible. We provided three examples of CiTo-3DP methodology use without fully describing its potential in different fields of research and education. However, we envisage that, in the future, the proposed CiTo-3DP methodology could be utilized for a variety of applications, including (but not limited) to in silico simulations for biology, medicine, pharmacological research, tissue engineering, morphometrical analysis, multiphysics modeling, education, rehabilitation of visually impaired people, and integration into virtual reality.

## 5. Conclusions

In conclusion, the presented CiTo-3DP approach bridges the gap between the high-resolution imaging of subcellular living structures and additive manufacturing, allowing the translation of cellular biology messages through tactile accessible, and interactive 3D printed models, and providing educators and researchers with a new way to display and analyze complex biological and engineering data.

## Figures and Tables

**Table 1:** Morphometric characteristics of the modeled cells.

Cell Type	Morphometric Characteristics, Mean ± St. Dev., µm				
	Cell Body, D1	Cell Body, D2	Nuclei, D1	Nuclei, D2	Nuclei, D_avg_
Epithelial (PANC-1)	44.6 ± 6.2	35.9 ± 3.8	18.8 ± 2.3	13.7 ± 0.8	16.2 ± 3.2
Mesenchymal (HDF)	195.5 ± 70.5	43.4 ± 13.4	44.1 ± 25.3	17.2 ± 4.7	N.A.
Neuron-like (SH-SY5Y)	16.5 ± 0.9	9.7 ± 2.0	6.4 ± 0.8	5.9 ± 0.3	6.1 ± 0.6

Abbreviations: St. Dev.—standard deviation; D1—longest diameter; D2—shortest diameter; D_avg_—average diameter; N.A.—not applicable.

## Data Availability

The relevant data generated and used in the current study are available from the corresponding author upon reasonable request.

## Appendix A

**Table A1:** The 3D-printed models developed on the basis of microscopic images of biological objects (literature analysis).

AM Parameter	Biological Object					
	Natural Killer Cell	Blood Cells	*C. elegans* Embryo and Distal Tip Cell, a Part of a Plant Cell	Pollen, Blood Cells, Plant Root, Insect Eye, Zebrafish Larva	Nucleus of a Hela Cell, Pollen Shells, Fruit Fly	Plant Golgi Stacks
References	[4]	[21]	[20]	[5]	[22]	[23]
Imaging modality ^1^	STED	TEM	MPM, SCF, and TEM	SCF (all samples, except zebrafish larva) and LSHM (zebrafish larva)	SCF	TEM
Detected signals ^2^	Phalloidin-Alexa Fluor 488 (f-actin)	SET	AF, gGFP	AF (all samples, except zebrafish larva), Phalloidin-FITC (f-actin), DAPI (omitted) * (zebrafish larva only)	DAPI (cell nucleus), AF (other objects)	SET
3D reconstruction	Huygens, Imaris, Blender	AMIRA, IMOD, Rhinoceros 3D	Mimics, Geomagics	Bitplane Imaris, MeshLab	ImageJ 3D Viewer Plugin	IMOD, 3dmod
3D printer	MakerBot Replicator 2X	FELIX 3.0 Dual Extruder	Spectrum Z510	Zortrax M200	UP Plus 2	Lulzbot Mini and Form1+
Printing software ^3^	Makerware	Repetier-Host for FELIX Printers	Zprint Software	Zortrax Z-Suite	N.D.	N.D.
3D printing technology ^4^	FDM	FDM	IPT	FDM	FDM	FDM and SLA
Printing material ^5^	ABS filament	PLA, PVA (support)	Plaster Powder	ABS filament	TP	TP and UR

Abbreviations: AM—additive manufacturing; ^1^: STED—Non-diffraction limited stimulated emission depletion microscopy, TEM—transmission electron microscopy, MPM—Multiphoton microscopy, SCF—scanning confocal microscopy, LSHM—lightsheet microscopy; ^2^: SET—serial electron tomography; AF—autofluorescence, gGFP—genetically encoded green fluorescent protein, Phalloidin-FITC—phalloidin conjugated with fluorescein isothiocyanate; DAPI—4′,6-diamidino-2-phenylindole, DAPI (omitted)*—zebrafish larva also was stained with DAPI, however, in the final visualization and 3D printed model the nuclear staining was omitted; DNA—desoxyribonucleic acid; ^3^: N.D.—no data provided; ^4^—FDM—fused deposition modeling, IPT—inkjet printing technology, SLA—stereolithography; ^5^—ABS—acrylonitrile butadiene styrene, PLA—polylactic acid, PVA—polyvinyl alcohol, TP—thermoplastic (unspecified), UR—unspecified resin.

### Appendix A.1. The Biomedical Educational and Research Context

The link between education and AM has previously been made by Kaplan et al. [32] and others, showing learning outcomes of complex concepts are greatly improved through the production of physical data visualizations. Further to this, education is made more accessible and comprehensible to special needs and disabled students. Similar conclusions have been drawn in cell biology and microscopy-related fields by Perry et al. [5]. In these fields, image data are often visualized three-dimensionally digitally, on analytically powerful software such as ImageJ [30]. Visualizing data in this way is effective but requires additional cognitive load to isometrically decode within the human brain. Three-dimensional printing data reconstructions effectively reduce this cognitive load, and as such, has the capacity to revolutionize how scientists analyze and present their results and findings.

In biomedical and bioengineering education, the presented CiTo-3DP methodology may be used to improve learning outcomes associated with complex biological concepts, such as cell morphology and tissue development. Additive technologies can provide biological constructors consisting of cellular and subcellular parts which can become a great way to teach spatial concepts such as tissue architecture and intracellular compartmentalization.

The CiTo-3DP method goes beyond the teaching of “generic” eukaryotic cell types by showing the obvious variety in cell morphometry between PANC-1 and HDF cells (Figure 5). As an example, students could be led through a laboratory-based experiment to culture and image basic mammalian cell phenotypes from epithelial, mesenchymal, or endodermal tissue origin. Other complex concepts such as cancer pathogenesis, cell shape regulation, EMT/MET, fibrosis, cell phenotypes, and transdifferentiation, as well as the general concept of dimensionalities—can be explored in specially designed experiments that now can be enhanced with 3D tactile visualization.

Students could then develop their own interactive models in image processing compatible CAD software and 3D print them using FDM printers located at their respective institutes. Such a workflow would provide students with hands-on experience in cell culturing, microscopy imaging, computational data analysis, and CAD, whilst also providing them with enhanced learning outcomes.

We suggest that some aspects of the presented CiTo-3DP protocols are particularly relevant for the biological and biomedical education and research context.

- Cell culture terminology and methodology: linear (immortalized) cells vs. primary cells [33]. The technical article by Merck explains cell culture protocols applicable both to linear cells (presented by cancer PANC-1 cells) and primary cells (human dermal fibroblasts, HDF).
- Healthy cells (HDF) vs. cancer cells (PANC-1). Fibroblasts are the main cell type in connective tissues, responsible for the production and degradation of collagen and other components of the extracellular matrix. A specialized form of fibroblasts, myofibroblasts, can exert strong contraction of tissue (particularly important for wound healing and regeneration). The source of PANC-1 cells in pancreatic ductal adenocarcinoma is deadly cancer with limited treatment options [34,35]. This malignant tumor commonly contains large amounts of collagen and fibroblasts, which together contribute to its treatment resistance [36].
- Embryonic origin of cells and tissues. Pancreatic adenocarcinoma originates from pancreatic glandular epithelium which has an ectodermal embryonic origin. Fibroblasts are cells of mesodermal origin. Neuron-like cells SH-SY5Y are derived from human neuroblastoma, a malignant tumor that originates from the neural crest cells.
- Cell shape and phenotype. Untreated PANC-1 cells are characterized by epithelioid phenotype; the HDFs have a mesenchymal-like phenotype, while SH-SY5Y cells may have varying phenotypes, depending on the cell culture conditions. In the current study, neuronal-like differentiation was maintained in these cells. Among several classifying features, morphology is one of the most prominent and obvious signatures of cellular phenotype. Epithelioid cells have a rounded shape, and their nucleus is usually centrally located. The signature of mesenchymal cells is a more elongated shape, quite often spindle-like, and the nucleus of the cell is usually more eccentric [33]. Neurons feature a clearly discernible cell body with centrally located round nuclei and various types of cytoplasmatic processes (the branching ones are termed dendrites, and the long, non-branching processes are named axons). Cell shape is a recognized feature associated with adhesion and motility potential, as well as their differentiation commitment [37,38,39].
- EMT and MET. One of the critical hallmarks of cancer progression is the so-called epithelial-to-mesenchymal transition (EMT) and the reverse (MET) process, reflecting the adaptation of cancer cells to new environments, for example, during the metastatic colonization of distant organs. The signature for EMT is a loss of epithelioid phenotype in epithelial (healthy or malignant) cells and the acquisition of a mesenchymal phenotype. MET presents the opposite transition. EMT/MET phenotype changes are reflected, in particular, in cell shape [40,41].
- Fibrosis. Fibrosis is the scarring of tissues and organs, characterized by excessive accumulation of extracellular matrix. At certain stages, it is also associated with the rapid proliferation of fibroblasts and their transformation into myofibroblasts. The signature of myofibroblasts is an expression of α-smooth muscle actin (α-SMA). The fibroblast-to-myofibroblast transdifferentiation, as well as the transformation of other cells into myofibroblasts, is a typical sign of fibrosis [42,43,44,45,46].
- Cytoskeleton. Three types of subcellular structures were imaged in the current study: cell nuclei, polymerized f-actin filaments representing the cytoskeleton component defining the shape of cells [37,38,39], and the specialized form of actin, known as α-smooth muscle actin (α-SMA), which is recognized as a phenotypical marker of cells bearing mechanical stress, such as smooth muscle cells or myofibroblasts [47]. Both the shape of the cytoskeleton and the level of α-SMA expression are key indicators of the cell’s functional state. Cell cytoskeleton and nucleus shape are dynamic characteristics that can reflect the phase of the mitotic cycle and the migration pattern [48]. In standard two-dimensional cell culture models, larger mean surface area and proportion of contractile α-SMA fibers indicate myofibroblast transdifferentiation of fibroblasts followed by excess synthesis of collagen [49]. This phenotypical transition reflects cellular fibrotic response on the tissue level, e.g., in skin scar or peri-implant connective tissue capsule formation [50]. These parameters are important to monitor in in vitro studies of cancer treatment, drug testing, and all the areas where cells are responding to external factors.
- The research and bioengineering applications of the CiTo-3DP methodology are dependent on the choice of the cells and subcellular structures. For the printed cellular models presented in this study, we envisage a scope of analytical tasks related to the relationship between the nucleus and cytoskeleton. For example, the data on the mass vs. the volume of the organelles, the surface texture of the organelles, the architecture of the intracellular space, and their reorganization in response to the experimental stimuli could serve for more biologically accurate bioengineering simulations such as, for instance, computational fluid dynamics research and analysis of the intracellular mechanical microenvironment. Further development of the proposed approach with the development of multi-material models or layered multi-material coatings, may be useful in cognitive and rehabilitation sciences.

**Table A2:** Confocal microscopy image acquisition settings.

Settings	Subcellular Structures		
	Nuclei (DNA)	F-Actin	α-SMA
Staining	DAPI	TRITC	Alexa Fluor 647
Excitation laser, nm	405	561	640
Emission Filter (range), nm	430–470 (DAPI)	572–612	670–770
Excitation maximum	357	552	658
Emission maximum, nm	463	576	675

**Table A3:** Parameters of the confocal microscopy images.

Settings	Cell Type		
	PANC-1	HDF	SH-SY5Y
Image Size (pixels)	1024 × 1024	1024 × 1024	703 × 880
Objective	UPLSAPO 60×	UPLSAPO 20×	UAPON 100 × OTIRF 100×
Numerical Aperture	1.35	0.75	1.49
Immersion media	PBS	PBS	PBS
Image Size (XY) (µm)	212.132 × 212.132	636.396 × 636.396	87.380 × 109.381
Voxel resolution (XYZ) (µm/pixel^3^)	0.207 × 0.207 × 1.000	0.621 × 0.621 × 0.500	0.240 × 0.240 × 0.500
Number of z-slices	16	33	36

**Table A4:** Printing resolution.

Parameters	Cell Type	
	PANC-1, SH-SY5Y	HDF
Printing scale, %	100 (colored) and 200 (white)	100
Print scale (µm: mm)	1 (colored) and 0.5 (white)	1
Layer height (mm)	0.2	0.2
Resolution (µm)	0.1	0.2

## Appendix B

### Appendix B.1. Detailed Protocol Notes

- In the proposed workflow, 2D confocal microscopy image z-stacks were spliced together using GV-intensity thresholding in Mimics Research 21.0 software. PANC-1, HDF, and SH-SY5Y cells were separately cultured and stained for immunofluorescent visualization of nuclei and cytoskeletal structures under a confocal laser scanning microscope. Respective .tiff z-stack files and corresponding image .txt metadata files were created as follows: 16 PANC-1 culture XY-planar images were sequentially captured and stacked under 60× oil-submerged magnification, generating an XYZ voxel resolution of 0.207 × 0.207 × 1.000 (µm/pixel3). Similarly, 33 HDF culture XY-planar images were sequentially captured and stacked under 20× magnification, generating an XYZ voxel resolution of with XYZ voxel resolution of 0.621 × 0.621 × 0.500 (µm/pixel3). Similarly, the imaging parameters for the SH-SY5Y cells are shown in Table A3 (Appendix B). All sets of images were optimized by adjusting laser intensity and voltage gain and offset of the PMT-amplified signal.
- Note that voxel resolution was identified as the principal determinant of image quality and hence 3D reconstruction accuracy.
- The open-source Ultimaker software CURA v4.7.0 was used to prepare the .stl files for 3D printing and configure the print settings of the selected printer. All cell types were printed simultaneously on a dual-extrusion Ultimaker S5 FFF-technology printer with a 330 × 240 × 300 mm build volume.
- White PLA material was extruded at 205 °C through a 0.4 mm extruder head onto a build plate surface at 65 °C. Fast printing settings were chosen to minimize printing time, which came to approximately 12 h. In particular, a 10% infill and 60° support angle were chosen. The printer was allowed to cool prior to removing the printed models from the build plate. Supports were removed by hand and with the aid of plyers.
- Cropped 3D reconstructions of cellular structures were generated in Mimics Research 21.0 software from the imported z-stacks using grey-value thresholding. Single PANC-1 and SH-SY5Y cells and two connected HDF cells were, respectively, isolated. Minor edits were made to masks to better represent the cellular components imaged. Specifically, the Smart Fill tool was used to fill small holes between reconstructed voxels.
- This was particularly important in generating close-to-solid nucleus structures. The initial length measurement of a selected nucleus object was taken in µm for scale verification throughout the workflow. The resultant objects were exported directly into Mimics 3-Matic for post-processing. A second length measurement of the previously selected nucleus object was taken in mm, which verified the import rescaling from µm to mm automatically performed by the Mimics software.
- The 3D objects were optimized for 3D printing using various editing tools. A 1 mm external uniform offset was applied to the meshed surface geometries, followed by iterations of the smoothing, wrapping and remeshing tools. The models were designed such that the nucleus could be extracted from the rest of the cell body model. To achieve this, an XY-plane trim was performed to slice the cytoskeleton geometry in half. The aligned nucleus geometry was then Boolean-subtracted with a 1 mm clearance factor from the trimmed cytoskeleton geometries.
- Finally, the quality of the resultant surface meshes was checked using the Fix Wizard tool. The surfaces (nucleus, cytoskeleton upper, cytoskeleton lower) were exported as separate .stl files. Mimics and 3-Matic (Materialise) are already readily used in biomedical research applications as design-orientated software.
- As previously noted, Mimics have been used by Liu et al. [14] to improve surgical planning and performance, as well as by McMenamin et al. [13] as a cheaper and more ethically neutral alternative teaching aid to cadavers in medical education. In comparison to other commercial software, Martin et al. [7] showed that Mimics possessed more powerful image manipulation, visualization, and editing functions. 3-matic, also part of Materialise and often packaged with Mimics, allows for further design iterations and is well-suited to optimizing meshes for FDM 3D printing as STL files.
- Notably, neither software has seen significant uptake in areas of micro-scale biology. In comparison to open-access image-processing software such as 3D Slicer and ImageJ, commercial software provides a faster, more powerful, and more versatile user experience. In terms of CAD, commercial software, such as 3-Matic, allows for greater interactivity to be easily built into printable models. Although this design power was not fully explored in this methodology, its effect was demonstrated by the interactivity of the PANC-1 cytoskeleton-nucleus cell model. To achieve similar results using free software would require a transfer between software, which is often cumbersome in terms of file formatting and file sizes. Considering that Materialise provides both image-processing and CAD, and is already used in biology-related sciences, it was chosen for this project. It should be noted that Materialise also offers a variety of online tutorial resources, making it far easier to learn the software.
